# A Bioinspired Multi-Level Numerical Model of the Tibiofemoral Joint for Biomechanical and Biomimetic Applications

**DOI:** 10.3390/biomimetics10020119

**Published:** 2025-02-18

**Authors:** Yuyang Wei, Yijie Chen, Sihan Jia, Lingyun Yan, Luzheng Bi

**Affiliations:** 1 Department of Engineering Science, University of Oxford, Oxford OX1 3PJ, UK; yuyang.wei@eng.ox.ac.uk; 2 Department of Mechanical, Aerospace and Civil Engineering, University of Manchester, Manchester M13 9PL, UK; 3 Department of Civil Engineering, University of Birmingham, Birmingham B15 2TT, UK; 4 Department of Robotics Engineering, School of Electrical and Electronic Engineering, Shanghai Institute of Technology, Shanghai 201418, China; 5 School of Mechanical Engineering, Beijing Institute of Technology, Beijing 100811, China

**Keywords:** biomechanics, finite element model, tibiofemoral joint, cartilages, meniscus, tibiofemoral ligaments

## Abstract

This study presents a comprehensive three-dimensional finite element (FE) model inspired by the biomechanics of the human knee, specifically the tibiofemoral joint during the gait cycle. Drawing from natural biological systems, the model integrates bio-inspired elements, including transversely isotropic materials, to replicate the anisotropic properties of ligaments and cartilage, along with anatomically realistic bone and meniscus structures. This dual-material approach ensures a physiologically accurate representation of knee mechanics under varying conditions. The model effectively captures key biomechanical parameters, including a maximum medial tibial cartilage contact pressure of 16.75 MPa at 25% of the stance phase and a maximum femoral cartilage pressure of 10.57 MPa at 75% of the stance phase. Furthermore, its strong correlation with in vivo and in vitro data highlights its potential for clinical applications in orthopedics, such as pre-surgical planning and post-operative assessments. By bridging the gap between biomechanics and bioinspired design, this research contributes significantly to the field of biomimetics and offers a robust simulation tool for enhancing joint protection strategies and optimizing implant designs.

## 1. Introduction

The human knee joint is a crucial diarthrodial joint that supports most of the body’s weight during gait. It serves as a biomechanical marvel, inspiring bioinspired designs in biomechanics and robotics. The mechanical properties of the tibiofemoral joint are essential in understanding knee function, improving joint protection strategies, and providing a theoretical basis for developing advanced knee implants and other bioinspired technologies. Finite element (FE) modeling is a versatile tool that allows researchers to simulate the contact behavior and kinematics of native or abnormal joint motions, offering insights that bridge the gap between biomechanics and biomimetics. Articular cartilage degeneration, such as osteoarthritis, is a leading cause of disability and orthopedic diseases [1]. Physiological factors, including age, sex, injury, and disease, significantly influence tibiofemoral joint motion. Among knee ligament injuries, 90% involve the anterior cruciate and medial collateral ligaments [2,3,4,5,6]. To address this, four primary tibiofemoral ligaments—the anterior and posterior cruciate ligaments, and medial and lateral collateral ligaments—along with the patellar tendon, were represented as solid models in this study to ensure anatomical and biomechanical accuracy.

While previous FE models of the knee joint have contributed to the field, most focus on static analysis and fail to produce real-time data during gait [7,8,9,10,11,12,13]. Additionally, many models use 1D spring elements or 2D shell elements to represent the tibiofemoral ligament, which inadequately captures the complex geometry and mechanical properties of soft tissues [14]. This study employs advanced imaging techniques, such as MRI and DICOM data, coupled with reverse engineering, to reconstruct detailed ligament and cartilage geometries. To model the mechanical behavior of these tissues, isotropic hyperelastic neo-Hookean materials were utilized, addressing the limitations of earlier models that neglected ligament anisotropy [15,16].

The neo-Hookean model was selected for soft tissue modeling due to its ability to capture the primary nonlinear elastic response of ligaments and cartilage under physiological loading while maintaining computational efficiency and numerical stability. Compared to complex hyperelastic models such as Ogden, Mooney–Rivlin, and Yeoh, which require multiple parameters that must be experimentally calibrated, the neo-Hookean model uses only two material parameters (initial shear modulus and bulk modulus), reducing uncertainties in parameter fitting. Additionally, high order hyperelastic models can introduce computational instability and higher computational costs, making them less practical for large-scale finite element simulations. Prior studies have validated the neo-Hookean model for knee joint biomechanics and ligament mechanics, demonstrating its suitability for capturing tissue behavior in a computationally efficient manner. While alternative models may offer greater accuracy for extreme deformations, the neo-Hookean model provides a robust and efficient approach for multi-level knee joint simulations, with future refinements potentially incorporating subject-specific parameter calibration.

FE modeling of the tibiofemoral joint plays a crucial role in various biomedical applications. While many existing models focus on orthopedic applications, such as prosthetic design and surgical planning, the relevance of biomechanical modeling extends further into rehabilitation, sports biomechanics, and injury prevention. For example, accurate simulations of joint dynamics can aid in the development of assistive devices for patients with musculoskeletal disorders, optimize rehabilitation strategies for post-surgical recovery, and inform injury prevention techniques for athletes. Furthermore, advancements in bioinspired computational modeling contribute to the development of smart wearable exoskeletons and personalized rehabilitation programs, enhancing patient-specific treatment approaches. By integrating these broader applications, our study provides a versatile computational framework that benefits multiple disciplines beyond orthopedics Several studies have investigated finite element modeling of the tibiofemoral joint, with a focus on stress distribution, ligament function, and contact mechanics. Traditional models, such as those developed by [17,18], simplify joint geometries and assume homogeneous material properties, limiting their applicability in realistic biomechanical simulations. More recent work by [19] has incorporated anisotropic properties and patient-specific data, but challenges remain in integrating hierarchical features. Additionally, studies by [20,21] have explored knee joint dynamics under different loading conditions; however, these approaches often lack the ability to capture microstructural interactions within the joint. These limitations highlight the need for a bioinspired multi-level numerical model that accurately represents soft tissue mechanics and dynamic responses during gait.

Existing finite element models of the tibiofemoral joint often rely on simplified geometries and homogeneous material assumptions, limiting their ability to capture the complex biomechanical interactions within the joint. These limitations hinder the accurate prediction of stress distribution, ligament function, and contact mechanics under dynamic loading conditions. Furthermore, while modeling has been explored in various biomechanical studies, its application to knee joint simulations remains underdeveloped. To address these challenges, this study introduces a bioinspired multi-level numerical model that integrates anatomically realistic structures and hierarchical material properties, offering improved accuracy in biomechanical analysis. This approach bridges the gap between conventional joint modeling and biomimetic applications, enabling advancements in personalized medicine, prosthetic development, and orthopedic treatments.

Our proposed multi-level modeling technique integrates body-level and tissue-level simulations, providing a comprehensive platform for advanced clinical diagnostics, particularly for patients with osteoarthritis or those requiring knee implants. In vivo and in vitro studies have been essential in validating knee joint biomechanics [22,23,24,25]. For instance, Liu et al. [22] conducted an in vivo study to evaluate tibiofemoral cartilage contact biomechanics during normal gait for eight healthy subjects, providing reference values comparable to our FE model [22]. These studies underscore the importance of experimental validation and boundary condition calibration for FE simulations. 

## 2. Materials and Methods

A three-dimensional and subject-specific FE model was reconstructed by applying a reverse engineering technique to evaluate biomechanical parameters (e.g., contact stress and cartilage deformation) throughout the full stance phase. The experimental results from Liu et al. [22] were used as the kinematic motions of the body-level model; this motion was captured using magnetic-resonance-scanned images combined with a dual fluoroscopic system during gait cycles on a treadmill; the output muscle force and joint contact load of the skeleton model were treated as boundary conditions of the subsequent tissue-level simulations.

### 2.1. Body Level Simulation

The body-level simulation aimed to determine the tibiofemoral joint contact load (bone-on-bone load) and individual muscle forces during the stance phase of a specific subject, which were then set as boundary conditions for the FE model. The simulation was conducted using OpenSim (National Centre for Simulation in Rehabilitation Research, Stanford University), and four key muscle forces—adductor magnus, lateral and medial gastrocnemius, and quadriceps—were derived, as they significantly influence femoral motion and joint loading. To ensure anatomical accuracy and physiological relevance, the modeling framework integrated high-resolution imaging data, biomechanical constraints, and material property variations, enabling a more precise representation of dynamic joint behavior. This approach improves upon prior finite element models by capturing the complex interactions of soft tissue mechanics and ligamentous constraints, reducing oversimplifications commonly seen in previous studies.

The OpenSim Gait 2392 model containing the trunk and legs was used for the body level simulation. The Gait 2392 model has 92 muscle-skeleton actuators representing 76 muscles in the lower extremities and torso, with 23 degrees of freedom at the joints. The length, mass, and inertial properties of the generic model were scaled according to the motion data captured by Liu et al. [22], and multiple walking speed simulations were performed using the motion data collected from six subjects. The scaled models and walking motion data for all subjects are available from Simtk.org (https://simtk.org/projects/mspeedwalksims, accessed on 5 September 2022). The motion data for Subject 6, an 81.9 kg female with 0.8 m-long legs, were chosen for the body level simulation. The motion data from six subjects was selected to balance computational efficiency and intersubject variability. Prior studies [17,18,19] have shown that a limited yet representative subject pool provides reliable gait insights while maintaining feasible computational demands. This sample size captures key knee joint dynamics while minimizing inconsistencies from anatomical differences, aligning with established multi-level biomechanical modeling research.

**Figure 1:**
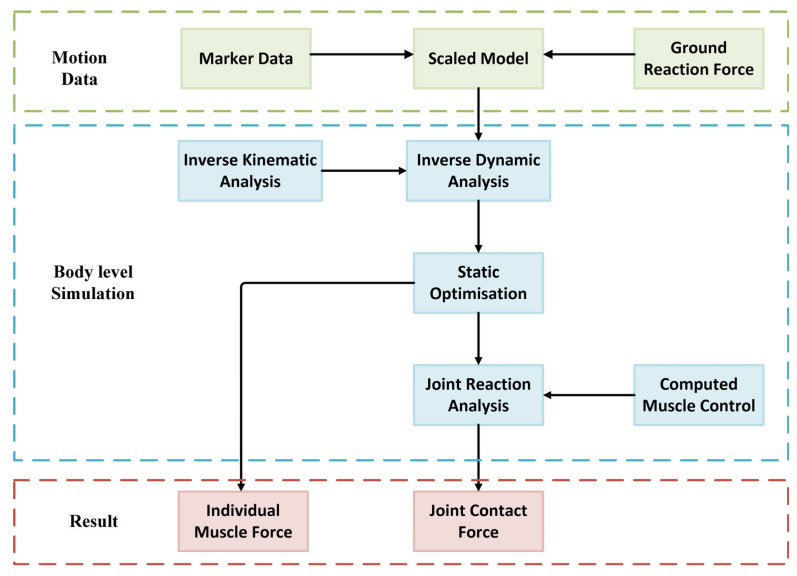
Body level simulation procedure.

The motion data includes the coordinates of the markers on each segment through the whole gait cycle and the ground reaction force under a walking speed of 1.11 m/s. The walking speed was chosen based on prior gait studies, aligning with the typical self-selected velocity in healthy adults [26,27]. Normal walking speeds range from 1.1 to 1.4 m/s, ensuring physiological relevance and consistency with validated biomechanical models. The subject (1.78 m, 68.95 kg) represents an average adult male based on anthropometric data [28,29] providing generalizable results while minimizing computational complexity. This selection balances accuracy and efficiency, with future studies incorporating broader anthropometric variations for sensitivity analyses and personalized modeling. The general body level simulation procedure in OpenSim is shown in the flowchart in Figure 1.

As shown in Figure 1, an inverse kinematic and dynamic analysis was carried out to generate the joint angle, coordinate, and contact force, which excluded the contributions of muscles. Static optimization was then applied to derive the individual muscle forces, which were combined with the muscle control simulation results for joint reaction analysis to calculate the joint resultant load. The individual muscle forces and tibiofemoral contact load were obtained from the static optimization and joint reaction analysis results, respectively, and are shown in Table 1.

### 2.2. Geometric Reconstruction

**Figure 2:**
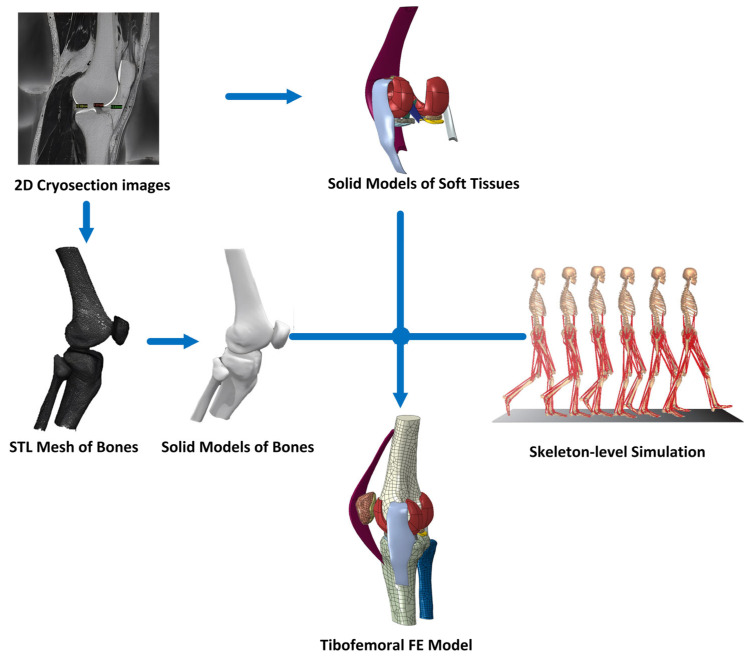
Main research procedure.

First, 424 slices from the “Visible Human Project” were extracted and processed using Mimics to create a 3D mesh in STL format. The tibiofemoral joint model was reconstructed from medical imaging data using a multi-step process. The Visible Human Project dataset was processed in Mimics software (Version 15), where manual segmentation was performed to extract relevant anatomical structures. The segmented data was then exported as STL files, which were further processed in PTC Creo 7.0 using its reverse engineering module to manually convert the surface mesh into a high-resolution 3D CAD solid model. This refined solid model was then prepared for finite element simulation to ensure smooth surface definitions and eliminate artifacts from the segmentation process. Although Mimics allows for high-precision anatomical reconstructions and seamless integration with FE analysis, it requires manual adjustments for soft tissue segmentation and is computationally intensive for large datasets. Due to the low contrast of ligaments in CT images, ligaments were not directly extracted from DICOM data but were manually reconstructed based on anatomical landmarks and validated using reference studies. This ensured anatomical accuracy in ligament placement within the FE model, with properties assigned based on experimental stress-strain data. The bones and meniscus were reverse engineered, and the ligaments were created from anatomical positions. Boolean calculations were performed to eliminate interference, resulting in the final geometry. The process is illustrated in Figure 2.

The static optimization process was employed to estimate the joint reaction forces while minimizing excessive computational costs. This method solves an optimization problem that distributes the net joint loads across different supporting structures (ligaments, cartilage, and contact interfaces) under equilibrium constraints. The optimization function aims to minimize physiologically unrealistic force magnitudes while ensuring dynamic consistency with the gait cycle [30,31]. Our approach follows established methodologies in biomechanical modeling, where static optimization provides a computationally feasible yet reliable approximation of joint loading conditions.

### 2.3. Tissue-Level Simulation

The FE model of the intact knee joint was developed using the developed solid model. The bio-mechanical behavior of tibiofemoral soft tissues (including meniscus and articular cartilage) during the stance phase is analyzed in this section. To achieve this, the FE solver Abaqus, which offers both pre- and post-processing capabilities, was employed.

To account for the varus valgus effect, we incorporated realistic anatomical structures and accurately captured the joint geometry in our FE model. This allowed us to simulate the natural range of motion of the tibia and femur, which includes varus and valgus movements. By enabling these articulations, we aimed to capture the varus valgus effect and its potential impact on the contact area and cartilage compression during the stance phase.

In previous FE analysis of the tibiofemoral joint, an explicit dynamic algorithm was found to be more suitable for simulating the dynamic loading of knee joints with relatively high-speed motion (from heel strike to toe-off) [13,32,33,34,35]. Carried out a comparative study on the outcomes of both implicit and explicit solutions in knee joint analysis [35] and found that the dynamic explicit simulation algorithm offers a significant reduction in computational time but similar predictions of cartilage stress and meniscus strains. Therefore, the dynamic explicit algorithm was chosen for this FE model. The whole simulation procedure was divided into six analysis steps, based on the five sub-steps of the stance phase (0%, 5%, 25%, 50%, 75%, 100% stance phase); the maximum step increment was set as 1 × 10^−6^ to provide accurate simulation results (because explicit algorithms are sensitive to the maximum increment). Nonlinear geometric control was used for each step because the ligament materials were hyper-elastic ones and as such can only be solved using the nonlinear algorithm in Abaqus/CAE. The linear and quadratic viscosity parameters remained at their default values of 0.06 and 1.2, respectively. We did not employ a preliminary step to explicitly ensure contact pressure greater than zero for the 0% stance phase. The reason is that our model considers the realistic geometry and kinematics of the knee joint during the entire gait cycle, and during the initial 0% stance phase, the contact forces between the tibia and femur are indeed minimal or close to zero due to the absence of weight-bearing.

#### 2.3.1. Material Properties

The material properties of bones, articular cartilage, patellar tendon, and meniscus were defined as linear elastic isotropic materials, following published experimental results; the material properties are listed in Table 2 [36,37,38,39].

According to previous research into knee joint modeling, the four main supporting ligaments (posterior and anterior cruciate ligaments and lateral and medial collateral ligaments) were solid-modeled [2,5,44,45,46]. The isotropic, hyperplastic material sections were assigned evenly on the solid ligaments.

In our FE model, we employed the neo-Hookean hyperelastic material model (the Weiss–Limbert Model for Ligaments) [16] known for its ability to handle large deformations and defined by a strain energy function dependent on the first invariant of the deformation tensor and the shear modulus, to simulate the nonlinear, nearly incompressible behavior of ligaments under load. The neo-Hookean model was selected for its balance between computational efficiency and accuracy under moderate strain conditions, which are typical in knee joint biomechanics. Unlike more complex hyperelastic models such as Ogden, Mooney–Rivlin, and Yeoh, it requires fewer parameters, reducing uncertainties in experimental fitting and improving numerical stability. This model has been successfully used in knee biomechanics studies for ligament and cartilage modeling. While it does not fully capture anisotropic and viscoelastic behavior, future refinements may integrate fiber-reinforced or poroelastic models to enhance physiological accuracy.

**Figure 3:**
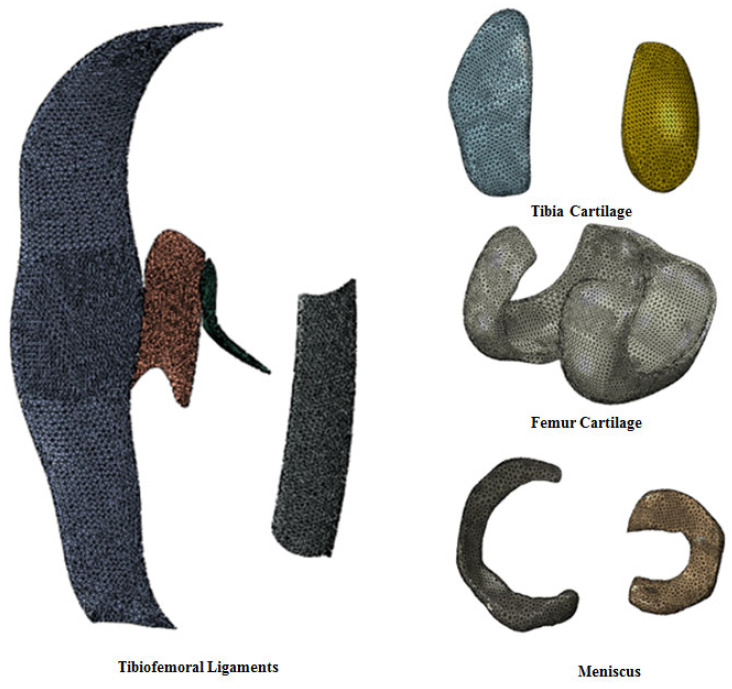
FE models of soft tissue.

The material properties for the present computational model were extracted from that model [16] The parameters of the neo-Hookean theory in the Weiss model are shown in Table 3 [35]. The FE models for soft tissues are shown in Figure 3. To enhance the realism and accuracy of ligament behavior, particularly under compression, we incorporated nonlinear spring elements designed to specifically react to compressive forces—a critical feature in joints where ligaments are subjected to both tensile and compressive stresses.

These springs exhibit variable stiffness characteristics that intensify with increasing compressive deformation, modeled through a nonlinear stiffness curve. Specific to the ligaments within the knee, nonlinear springs were applied with stiffness ratings adjusted for their biomechanical roles: the posterior and anterior cruciate ligaments (PCL and ACL) received springs scaling from 300 N/mm to 600 N/mm to reflect their function in stabilizing against hyperextension and hyperflexion, while the lateral and medial collateral ligaments (LCL and MCL) had springs ranging from 200 N/mm to 400 N/mm to support knee stability against varus and valgus stresses [4]. These elements were strategically positioned and oriented according to the anatomical ligament directions to ensure precise contribution to joint mechanics, interacting with surrounding cartilage and bone to influence load distribution and kinematics accurately within our model. The detailed definitions of the spring elements are listed in Table S1 of the Supplementary Material.

The transversely isotropic material model was implemented to capture the direction-dependent mechanical behavior of ligaments and cartilage. Material properties were assigned with a preferential fiber direction to reflect anisotropic stiffness and load-bearing capacity. The constitutive relation was defined using a neo-Hookean hyperelastic matrix combined with a fiber-reinforced component.

#### 2.3.2. Mesh Assignment

Most previously developed FE human knee joint models used tetrahedral elements for soft tissues and hexahedral elements for bones [10,11,46]. Several researchers have preferred hexahedral elements for meshing model components because tetrahedral elements can produce more severe distortions in dynamic analysis, potentially leading to errors during the analysis procedure [35,47,48]. However, due to the complex geometry of this tibiofemoral model, all segments were assigned the tetrahedral element ‘C3D4’. The sizes of the elements were determined via an element convergence test. We primarily focused on the element size for the C3D4 elements representing the articular cartilage. Our aim was to determine the minimum element size necessary to achieve convergence and obtain reliable results for the cartilage. In our case, when the element size is below approximately 1mm, the von Mises stress of the cartilage was found to differ by less than 5% from the results obtained with a two-element-thick (element size of 1mm) cartilage simulation. The results of the convergence test are presented in Figure S1 in the Supplementary Material. The finite element model consisted of 105,115 nodes and 482,286 elements, with a refined mesh applied to regions of high stress gradients, such as cartilage contact interfaces and ligament insertion sites.

#### 2.3.3. Loads and Boundary Conditions

**Figure 4:**
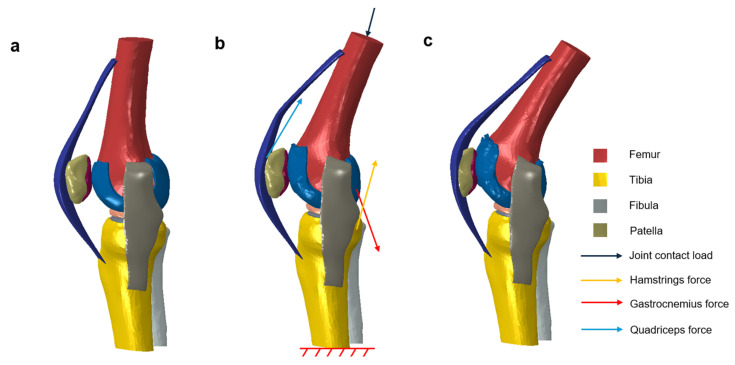
Solid model of the tibiofemoral joint during three different stance phases: (**a**) 10% stance phase, (**b**) 50% stance phase, with arrows indicating the muscle forces and joint contact load. The bottom of the tibia is fully fixed, as shown. (**c**) 100% stance phase.

Loads, including individual muscle forces and joint contact loads, were applied to the corresponding anatomical positions of the FE model based on body-level simulation results, as presented in Figure 4. The boundary conditions were determined from the kinematic data for the subject collected by Liu et al. [22], and the tibiofemoral joint angles captured during the stance phase were defined as the angular displacement required to simulate the kinematics of the tibiofemoral joint, as shown in Table 4; the cross-sectional plane on the distal part of the tibia was fixed.

The load application time was determined based on a combination of experimental data and biomechanical considerations. Specifically, we used a validated approach that considers the kinematics and forces acting on the joint during the gait cycle to determine the appropriate time to apply the load. The whole duration of the simulation is 0.6 s which corresponds to a normal duration of the stance phase. We utilized a time increment that balances computational efficiency and numerical stability. The specific duration of each sub-step was determined through iterative testing and validation to ensure reliable results. This phase of the gait cycle is known to involve critical biomechanical events and load distributions within the knee joint. By examining this specific phase in detail, we aimed to gain insights into the joint mechanics during a crucial transitional period. Additionally, studying the 5% stance phase allows for meaningful comparisons with existing literature and facilitates a comprehensive understanding of the joint behavior throughout the gait cycle. The femur, tibia, fibula, and patella were defined as rigid bodies. Seven surface-to-surface contact pairs were defined to represent the biomechanical contact behavior of the tibiofemoral joint [47]. We employed tie constraints to connect the fibula and tibia at their respective interfaces. To ensure the accuracy of our modeling approach, we referred to anatomical literature and imaging data to determine the appropriate contact surfaces and constraints for the fibula-tibia connection. The choice of tie constraints was based on their ability to accurately represent the mechanical behavior and interactions between the fibula and tibia. A preliminary stabilization step was set up in ABAQUS. This step involves a predefined field initialization, where the joint contact load is gradually applied over 0.2 s period to ensure the model reaches a stable equilibrium before the dynamic analysis. This approach effectively mitigates large dynamic effects and provides a more accurate representation of the physiological loading conditions during the initial phases of the gait cycle. This preliminary step ensures minimal dynamic effects at the start of the simulation, providing more accurate and reliable results throughout the stance phase.

For each pair of contacts, a frictionless and finite sliding formulation was applied, following Pena and others’ research into knee joints [11,15,49]; the fluid properties of soft tissues can accommodate most of the load during the short loading times involved in normal gait cycles; this produces a relatively low or negligible friction between tibiofemoral soft tissues. The connections between the cartilages and their attached bones, ligaments, or tendons (with bones) were represented as “Tie” constraints; this configuration corresponds to the biological behavior of human knee joints. The horn attachments of the meniscus were represented as linear springs following Noyes’s in vitro investigation of the human knee joint [50], the stiffness was determined using the modulus and cross-sectional area of the measured ligaments, and the lengths of the ligaments were defined using the average length extracted from Noyes’s experimental results [50,51]. Therefore, the stiffness was 2000 N/mm, each of the horns of the meniscus was represented by ten linear springs with a stiffness of 200 N/mm, and the connection points for the horn attachments were distributed on the tibia plateau and meniscus.

For the patellofemoral joint, it was defined with the patella articulating against the femoral groove, incorporating realistic contact mechanics to simulate both the contact forces and the kinematic behavior of the patella as it tracks along the femur during flexion and extension movements. The contact interactions were defined using a surface-to-surface contact formulation, which allows for the accurate simulation of contact pressures and distribution across the joint surfaces. This contact model considers the complex geometry of the patella and femoral groove, employing a frictional contact algorithm to replicate the natural sliding and rolling motion of the patella over the femur throughout the range of motion. This comprehensive approach to modeling the patellofemoral joint ensures that our simulations provide a realistic representation of joint mechanics, which is critical for investigating patellar tracking disorders and the effects of surgical interventions on patellofemoral biomechanics.

To simulate varus–valgus and internal–external rotation, the model allows for varus–valgus rotations ranging from −5 degrees (varus) to +5 degrees (valgus) to capture the medial and lateral stability of the knee. These rotations reflect the knee’s response to uneven ground surfaces and lateral forces that might occur during dynamic activities. Internal–external rotations were simulated from −10 degrees (internal rotation) to +10 degrees (external rotation). A detailed boundary condition setup is presented in Table S2 of the Supplementary Material. This range is critical for activities that involve pivoting movements, providing insights into rotational stresses that ligaments and cartilages undergo during sports or sudden directional changes. This comprehensive setup allowed our FE model to predict contact pressures, tissue deformation, and joint stability with high fidelity, aligning closely with observed biomechanical data. The sensitivity test was also conducted by varying the muscle forces and joint contact load. Detailed results are shown in Table S3 of the Supplementary Material.

## 3. Results

The contact mechanism between the cartilage and meniscus under all six loading conditions was analyzed. Previous experimental results have shown that articular cartilage damage or osteoarthritis may be related to the large shear or principal strain on the cartilage surface [39,52]; hence, the maximum strain contour plots of the tibia and femur cartilage were also examined in this study. The cartilage contact area and pressure were extracted, and these simulated results were validated using in vivo and other experimental results, to provide a theoretical background for further clinical case simulations with this model and improve our understanding of tibiofemoral cartilage contact pressures during the stance phase. The in vitro and in vivo results used for validation were obtained from previously published experimental studies. These datasets provide reliable benchmarks for joint contact pressure, ligament strain, and reaction forces, allowing for a robust comparison with simulation results. The agreement between our model and experimental findings supports the accuracy of the finite element framework.

### 3.1. History Output Results

Table 5 shows the contact area of the tibiofemoral cartilage. The maximum contact area of the medial tibia cartilage was 392 mm^2^ at a stance phase of 5% and 313 mm^2^ for the lateral tibia at a 25% stance phase. Furthermore, at an ~100% stance phase, a maximum contact area of 601 mm^2^ was captured in the femur cartilage. The contact area of the femoral cartilage was much larger than that of the tibial cartilage because the contact on the inferior part of the femoral cartilage includes contributions from both the medial and lateral tibial cartilages. Additionally, the femur was allowed to rotate freely above the tibia, further increasing the contact area.

The tibiofemoral pressure distribution simulation results are listed in Table 6. The maximum contact pressure on the medial tibia cartilage was 16.75 MPa at a 25% stance phase and 9.63 MPa at the lateral side; the femur cartilage also displayed 2.95 MPa at the beginning of stance phase. Both pressures on the medial and lateral sides of the tibia cartilage exhibited two peaks 25% of the stance phase, whereas the maximum pressure on the femoral cartilage was 10.57 MPa at a 75% stance phase and fell to 3.58 MPa at the end.

Table 1 shown previously illustrates the joint resultant loads, which are cumulative axial loads applied to the knee joint. These loads are a summation of forces acting on the joint, reflecting the overall mechanical demand during the gait cycle. In contrast, Table 6 details the maximal contact pressures, which are peak pressures at specific joint locations. These pressures are influenced by factors such as contact area, cartilage material properties, and the geometric distribution of forces. A critical observation in our study is the non-uniform distribution of forces across the knee joint’s articular surfaces. This non-uniformity can result in significant peak pressures at localized points, even under relatively low overall joint loads. It is crucial to understand that maximal contact pressures are not always directly proportional to the joint resultant loads due to this complex force distribution. Specifically, at the 50% stance phase, we observed a maximal compressive force that appeared relatively low. This can be attributed to the biomechanical characteristics of the knee joint during this phase of the gait cycle. The knee experiences a transition in load distribution, with changing leverage and muscle activation patterns. Consequently, this phase may not coincide with the peak stress points within the joint, leading to lower maximal compressive forces compared to other phases. Both joint resultant loads and maximal contact pressures provide essential insights into knee joint biomechanics. While the former offers a perspective on overall joint loading, the latter highlights areas subjected to high mechanical stress, crucial for understanding injury mechanisms and degenerative processes. This dual analysis approach is vital for a comprehensive understanding of knee joint function and pathology.

The tibial cartilage deformation was analyzed because previous research has shown that cartilage on the tibial plateau is more likely to degenerate and develop osteoarthritis. It was also found that tibia cartilage strain plays a significant role in articular cartilage degeneration; thus, the tibia cartilage deformation and strain are listed in Table 7 and help to validate the FE model against in vivo cartilage deformation results. As can be seen from Table 7, the maximum principal strains of the medial and lateral tibial cartilages exhibited the peak at 25% stance phase: 0.0845 and 0.0697, respectively. The deformation on the medial side was larger than that on the lateral side. The differences of the maximum strain between lateral and medial cartilage across the whole phase are shown in Table S4 of the Supplementary Material.

### 3.2. Field Output Results

As can be seen from Table 8, the contact pressure was mainly distributed on the medial tibial cartilage throughout the stance phase, and the maximum pressure was focused on the central part of the medial cartilage at the 25% stance phase. The pressure on the medial tibial cartilage exceeded that on the lateral side. The maximum deformation was also distributed along the medial-posterior edge of the medial cartilage during the stance phase, and the deformation of the medial cartilage exceeded that on the lateral side. 

According to previous research [53], the joint reaction load of the tibiofemoral joint is mainly transferred by the meniscus between the tibia and femur. Thus, the maximum contact pressure and deformation were displayed on the medial meniscus because the medial tibia cartilage underwent more stress and deformation than the lateral part. According to the simulation results in Table 7 and Table 8, another factor also contributes to the increased pressure on the medial meniscus: the anatomical position of the medial collateral ligament. This ligament was attached to the meniscus on the medial side whereas the lateral meniscus was only restrained by horn attachment and the transverse ligament, resulting in fewer degrees of freedom on the medial meniscus than on the lateral side. This finding corresponds with most clinical research results, which state that the medial meniscus has a greater chance of being torn or injured. A similar pressure and deformation distribution pattern was also observed on the femoral cartilage because the joint contact load and stress penetrated from the femur to the tibial plateau. Additionally, relatively low stress level was presented on meniscus. While the relatively low-pressure levels in the meniscus might suggest that it carries less load than expected, the low stress levels in the meniscus could be influenced by the chosen material properties, particularly in the context of the chosen hyperelastic model. A thorough sensitivity analysis was conducted by varying the material properties within a physiologically relevant range to validate its effects on the stress distribution. The results are presented in Table S4, the varied $E_{pt}$ and $E_{p}$ were adjusted from −50% to +50%, and the stress was significantly affected by the varied material properties. This analysis allows us to assess the impact of material properties on stress distribution in the meniscus. Additionally, a detailed analysis of the root mean square error (RMSE) between our simulation results and experimental results was conducted and is shown in Table S5 of the Supplementary Material. This shows that our predicted results align well with the experimental ones from the real world.

## 4. Discussion

The FE model developed in this study bridges the gap between biomechanics and biomimetics, serving as a platform for understanding joint mechanics and inspiring bioinspired technologies. Validation is a critical aspect of FE modeling, ensuring its reliability and application for future clinical and bioinspired studies. To ensure robustness, the stance phase simulation results were compared with Liu et al.’s in vivo and in vitro experimental data [22], as well as with other numerical models [7,8,9,10,11,12,13]. This comprehensive validation underscores the reliability of the proposed FE model.

### 4.1. Bioinspired Aspects

This FE model highlights several bioinspired features. The integration of anisotropic material properties for ligaments and cartilages replicates the natural mechanical behavior of knee tissues. Additionally, detailed geometries derived from advanced imaging techniques mimic the structural complexity of the human knee joint. Such biomimetic elements offer valuable insights into the design of knee implants, prosthetics, and robotic joints, aligning the study with biomimetics principles [15,16].

Traditional FE models of the knee joint often assume homogeneous material properties and simplified contact mechanics, limiting their ability to replicate real physiological behavior. The proposed bioinspired multi-level model improves accuracy by incorporating hierarchical tissue structures and anisotropic material definitions, allowing for more realistic stress distribution and load transmission. Unlike conventional static loading models, this approach integrates gait-driven force applications, enabling simulations of knee function under dynamic conditions. Additionally, by capturing soft tissue deformation and adaptive ligament constraints, it enhances joint contact mechanics compared to rigid or frictionless assumptions commonly used in traditional FE studies. These improvements provide better insights for biomechanical applications, including rehabilitation, exoskeleton design, and prosthetic development. Future refinements will further validate the model against experimental datasets to strengthen its applicability.

### 4.2. Material Strength in Relation to Stress Levels

The finite element analysis results indicate localized stress levels reaching 100 MPa and 80 MPa in specific regions of the tibiofemoral joint, particularly near subchondral bone and ligament attachment sites. These values align with reported ultimate tensile and compressive strengths of biological tissues, suggesting that high-stress areas may correspond to regions prone to mechanical overload. While cartilage and ligament tissues generally fail at lower stress levels, these concentrations highlight potential structural vulnerabilities under extreme loading.

### 4.3. Validation of Tibial Cartilage Contact Area

**Figure 5:**
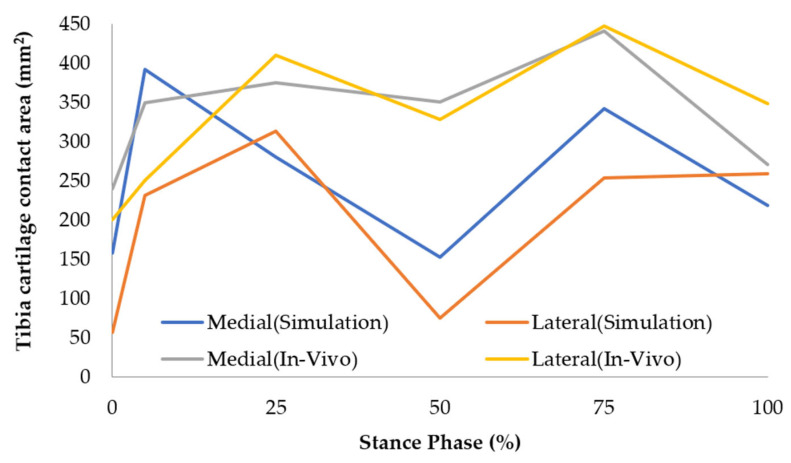
Validation of tibia cartilage contact area.

The contact areas between the femoral and tibial cartilage obtained from experimental results were compared with the simulation results of our FE model, as shown in Figure 5. The fluoroscopy technique used in the study by Liu et al. had limitations in capturing the tibiofemoral meniscus, potentially leading to an overestimation of the contact area [22]. Despite this limitation, Liu et al.’s study remains a benchmark for validating our model. Both the in vivo experiment and the FE simulation exhibited two peaks in contact area during the stance phase, observed at ~30% and ~80% in experimental results and 25% and 75% in the FE simulation 22. These data trends demonstrate good agreement, although the magnitudes differed slightly.

Factors contributing to these differences include variations in definitions of contact area, imaging limitations, material properties, walking speeds, and subject-specific variations. Specifically, Liu et al.’s study [22] defined the contact area as an overlapping region without considering cartilage deformation, which may have led to higher estimates. Additionally, the fluoroscopy technique could not capture the meniscus, which significantly influences load distribution, while our FE model accounted for meniscus mechanics. Our model’s use of linear elastic and transversely isotropic material properties contrasts with the inherently anisotropic, viscoelastic, and fluid-filled nature of articular cartilage [15,16]. Discrepancies in walking speeds and individual variations in proteoglycan content, which affects cartilage stiffness, further explain the differences [39].

### 4.4. Validation of Cartilage Deformation

**Figure 6:**
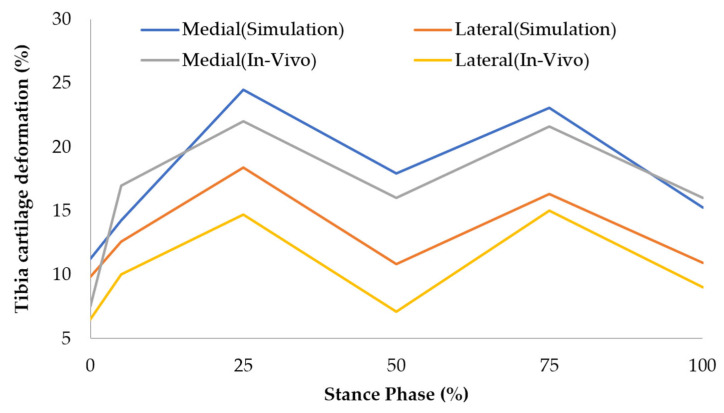
Validation of tibia cartilage deformation.

The deformation patterns from the FE simulation aligned well with in vivo data, as shown in Figure 6. Both datasets exhibited two deformation peaks during the stance phase at ~25% and ~75% [22]. The FE simulation showed medial tibial deformation surpassing lateral deformation, consistent with clinical findings that medial tibial cartilage accounts for 65% of the joint reaction load [54]. These results further validate the FE model’s accuracy in capturing realistic joint mechanics.

As shown in Table 9 [9,22], most experimental results indicate that the lateral tibial contact area slightly exceeded that of the medial side. Among these experiments, Hao et al. applied boundary conditions similar to those of the present computational model, and the cadaverous knee joint was compressed to 400 N with 15° flexion; this is similar to the boundary conditions used for the 25% stance phase in our tibiofemoral FE model. The differences between in vitro results and our FE results were less than 20% for the lateral side contact area and only 3% for the medial one [8]. The simulation results generally corresponded to the in vivo and in vitro experimental data; that is, the differences between the two sets of data were less than 33% and the results were of the same order of magnitude. Therefore, the FE simulation results in this project showed good agreement with the published in vivo and in vitro experimental results listed in Table 9.

The maximum principal strains derived from the FE simulation results are compared against the real test data in Table 10. The FE simulation results showed good agreement with the experimental data, which ranged from 3.5% to 9.5%. The small divergence between the numerical and experimental results is primarily due to the different strain-calculation algorithms used in the in vivo experiments, as the deformation of soft tissues was not fully accounted for. The large deformation of soft tissues during the normal stance phase may increase the contact area and strain of the cartilage; however, in the in vivo experiments, only the penetration distance into the cartilage was considered for soft tissue deformation. It can also be observed that the strain of the medial tibial cartilage exceeded that of the lateral cartilage in all in vivo experimental data, although the difference between the lateral and medial strains was relatively small compared to their magnitudes.

### 4.5. Potential Applications

The validated FE model has significant potential for biomimetic applications. It can inform the design of advanced prosthetics and joint implants, contributing to personalized treatment strategies in orthopedics. Furthermore, the model provides a foundation for developing humanoid robotic systems that mimic natural joint mechanics, showcasing the value of biomimetic approaches in addressing engineering and medical challenges [7,55].

### 4.6. Limitations of This Multi-Level Model

One limitation of this study is the reliance on motion data from Liu et al. [22], which may not fully capture subject-specific anatomical variations and dynamic adaptations. Differences in muscle activation, ligament laxity, and joint kinematics among individuals could introduce variations not explicitly accounted for. Additionally, the finite element model focuses on local joint mechanics, potentially overlooking whole-body dynamics such as compensatory movements at the hip and ankle during gait.

Environmental factors, including surface friction, incline, and external loads, were not considered, even though they can influence gait patterns and joint loading. The material properties used, while sourced from validated studies, may not fully reflect individual variability due to differences in age, health, and mechanical history. To balance computational efficiency, certain simplifications were made in modeling cartilage and ligaments, including assuming frictionless contact and linear elasticity, which may slightly affect contact stress predictions. Despite these limitations, the model effectively captures key knee joint mechanics and provides a strong foundation for further refinement. Future work will incorporate EMG-driven muscle forces, subject-specific material calibration, and adaptive tissue remodeling to enhance accuracy and real-world applicability.

### 4.7. Comprehensive Literature Review

This research builds on an extensive body of work, incorporating findings from studies on biomechanical modeling, biomimetic material properties, and in vivo validation methods. Key contributions from the literature were critically analyzed to contextualize this study within the broader field of biomimetics. The incorporation of detailed references ensures a thorough understanding of the current state-of-the-art and strengthens the foundation for future research and applications [7,8,9,10,11,12,13,14,15,16].

### 4.8. Application to Osteoarthritic Knee Models

While this study focused on the biomechanics of a healthy tibiofemoral joint, the FE model provides a solid foundation for simulating osteoarthritic conditions. Osteoarthritis introduces complex changes in joint structure, cartilage degradation, and altered biomechanics. To adapt the model for osteoarthritis, modifications such as incorporating patient-specific geometry, accounting for cartilage degeneration, and capturing altered joint contact patterns are necessary. These adaptations would enhance the model’s ability to simulate the wide range of disease stages and patient-specific variations associated with osteoarthritis. Additionally, the Supplementary Material (Tables S6–S8) highlights the role of the patella joint in reducing cartilage contact pressure and providing stability, emphasizing its relevance in both healthy and pathological conditions. 

This multi-level numerical model represents a significant advancement in the field of biomimetic biomechanics. Its validation against experimental and computational studies demonstrates its potential as a robust tool for clinical diagnostics and the development of bioinspired technologies. Future adaptations for osteoarthritic conditions will further expand its applicability, contributing to the advancement of biomimetics and personalized medicine.

## 5. Conclusions

This study developed and validated an FE model of the tibiofemoral joint, demonstrating high agreement with in vivo and in vitro experimental data. The model captured key biomechanical parameters, including a maximum medial tibial cartilage contact pressure of 16.75 MPa at 25% stance phase and a maximum femoral cartilage pressure of 10.57 MPa at 75% stance phase. The peak principal strain of the medial tibial cartilage reached 0.0845 (8.45%), while the lateral tibial cartilage exhibited a maximum strain of 0.0697 (6.97%). These results confirm the model’s accuracy in simulating joint mechanics and stress distributions. The validated computational framework provides a robust platform for future clinical applications, such as preoperative planning and implant optimization, and offers valuable insights for biomimetic joint designs.

## Figures and Tables

**Table 1:** Body level simulation results.

Stance Phase (%)	0	5	25	50	75	100
Adductor magnus (N)	17.53	98.35	47.59	6.11	6.67	9.74
Medial gastrocnemius (N)	110.84	82.77	73.15	378.63	1052.42	324
Lateral gastrocnemius (N)	27.49	15.95	27.73	82.6	223.93	77.06
Quadriceps (N)	13.21	264.99	763.07	118.25	145.98	225.28
Joint contact load (N), compressive	1008.32	497.56	756.02	1379.34	77.04	9.67
Joint contact load (N), shear	0.32	−78.45	−37.83	86.47	6.93	3.91
Joint resultant load (N)	1008.32	497.56	756.97	1382.05	77.35	10.43

**Table 2:** Material properties. Young’s modulus (*E*) and Poisson’s ratio (ν) are provided for different tissues. The subscript *p* denotes properties related to the proteoglycan matrix within soft tissues, while the subscript *pt* refers to the principal tensile direction, accounting for the anisotropic behavior of the meniscus and patellar tendon.

	Young’s Modulus	Poisson’s Ratio	Density	Techniques Applied
Cortical bone [40]	20,000 MPa	0.3	2000 g/cm^3^	Ultrasonic and micro-tensile
Articular cartilage [41]	*E* = 10 MPa	ν=0.3	1000 g/cm^3^	Confined compression testing
Meniscus [42]	$E_{p}$ = 20 MPa $E_{pt}$ = 140 MPa	$\nu_{p}$ = 0.2 $\nu_{pt}$ = 0.3	1000 g/cm^3^	Tonometer
Patella tendon [43]	$E_{p}$ = 30 MPa $E_{pt}$ = 300 MPa	$\nu_{p}$ = 0.45 $\nu_{pt}$ = 0.045	1000 g/cm^3^	Ultrasonic combined with MRI

**Table 3:** Parameters of the neo-Hookean theory in the Weiss model.

	Anterior Cruciate Ligament	Posterior Cruciate Ligament	Medial Collateral Ligament	Lateral Collateral Ligament
*C*_10_ (MPa)	1.95	3.25	1.44	1.44
*D*	0.00683	0.0041	0.00126	0.00126

where $C_{10}$ and *D* are temperature-dependent material parameters, and *D* controls the compressibility of the material. If *D* is equal to zero, the material can be regarded as impressible.

**Table 4:** Kinematic data for the in vivo experiment.

Stance Phase (%)	Flexion Angle (Degree)
0%	10.49
5%	13.18
25%	20.80
50%	12.26
75%	9.74
100%	24.29

**Table 5:** Contact area on femoral and tibia cartilages.

Stance Phase (%)	Tibia Contact Area (mm^2^)		Femur Contact Area (mm^2^)
	Medial	Lateral	Inferior
0	158	57	108
5	392	231	139
25	280	313	295
50	152	75	241
75	342	253	281
100	218	259	601

**Table 6:** Contact pressure on femoral and tibia cartilages.

Stance Phase (%)	Tibia Contact Pressure (MPa)		Femur Contact Pressure (MPa)
	Medial	Lateral	Inferior
0	4.55	3.41	2.95
5	7.95	7.52	5.83
25	16.75	9.63	9.84
50	8.73	3.74	5.96
75	15.84	8.74	10.57
100	3.07	7.97	3.58

**Table 7:** Tibia cartilage deformation and maximum principal strain.

Stance Phase (%)	Strain of Tibia Cartilage		Deformation of Tibia Cartilage (%)	
	Medial	Lateral	Medial	Lateral
0	0.0237	0.0198	1.24	2.15
5	0.0225	0.0228	4.27	2.57
25	0.0845	0.0697	9.25	5.92
50	0.0337	0.0350	7.94	4.85
75	0.0534	0.0495	8.56	5.25
100	0.0228	0.0195	3.95	2.84

**Table 8:** Contour plot of von-Mises stress on 25% stance phase.

Position	Contour Plot of Contact Pressure	Scale (Pa)
Femur cartilage	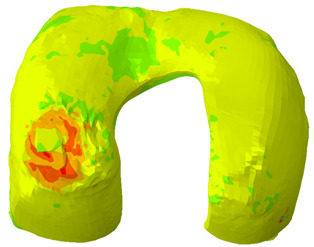	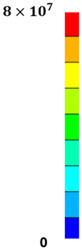
Meniscus	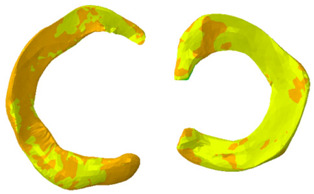	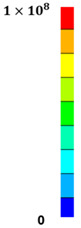
Tibia cartilage	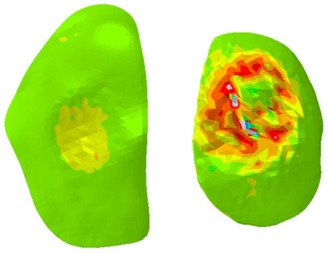	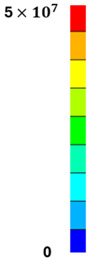

**Table 9:** Validation of tibia contact area with other in vivo or in vitro experiment.

Details	Medial Tibia Contact Area	Lateral Tibia Contact Area	Difference Compared to Simulated Results (Lateral/Medial)	Method	Experimental Type
Simulation results	342 mm^2^	313 mm^2^	/	FE analysis	Simulation
Liu et al. [22]	440 mm^2^	455 mm^2^	22%/30%	Fluoroscopy combined with MRI	In vivo
Hao et al. [9]	300 mm^2^	232 mm^2^	14%/35%	MRI	In vitro *cadaver*

**Table 10:** Validation of tibia deformation (maximum principal strain) against other in vivo or in vitro experiments.

Details	Medial Tibia Strain (%)	Lateral Tibia Strain (%)	Method	Experimental Type
Simulation results	9.3%	6.0%	MRI	Simulation
Hamai et al. [24]	3.5 ± 0.6%	3.4 ± 0.4%	MRI combined with solid-modeling software (SliceOmatic, Version 6)	In vivo
L. Coleman et al. [23]	4.9%	3.2%	MRI	In vivo
Smith et al. [25]	9.5 ± 2.4%	8.7 ± 1.0%	MRI	In vivo

## Data Availability

All the related data for this paper are provided in the Supplementary Materials with the publication.

## Supplementary Materials

The following supporting information can be downloaded at: https://www.mdpi.com/article/10.3390/biomimetics10020119/s1, Table S1: Detailed Definitions of Spring Elements in Ligaments and the load transfers; Table S2: Boundary Conditions for Varus-Valgus and Internal-External Rotation; Table S3: Results of sensitivity test; Table S4: Differences of the maximum strain between lateral and medial cartilage across the whole phase; Table S5: Root Mean Squared Error (RMSE) between our simulation results and experimental results; Table S6: The maximum stress on meniscus with different Young’s Modulus; Table S7: Contact area on femoral and tibia cartilage (without patella joint); Table S8: Contact pressure on femoral and tibia cartilage (without patella joint); Figure S1: Size for all the tissues were modulated together with the cartilages.
