# Supplementary material for: A Bioinspired Multi-Level Numerical Model of the Tibiofemoral Joint for Biomechanical and Biomimetic Applications
Source: Biomimetics (Basel). 2025 Feb 18;10(2):119. doi: 10.3390/biomimetics10020119 (PMC11853415; doi:10.3390/biomimetics10020119)
Supplement: Supplementary file 1 [file biomimetics-10-00119-s001.zip › biomimetics-3428898-supplementary.pdf]

Supplementary Information for

A Bioinspired Multi-Level Numerical Model of the Tibiofemoral Joint for Biomechanical and Biomimetic Applications

Yuyang Wei [1,2], Yijie Chen [2], Sihan Jia [3], Lingyun Yan [2,4,*] and Luzheng Bi [5,*]

1 Department of Engineering Science, University of Oxford, Oxford OX1 3PJ, UK
2 Department of Mechanical, Aerospace and Civil Engineering, University of Manchester, Manchester M13 9PL, UK
3 Department of Civil Engineering, University of Birmingham, Birmingham B15 2TT, UK
4 Department of Robotics Engineering, School of Electrical and Electronic Engineering, Shanghai Institute of Technology, Shanghai 201418, China
5 School of Mechanical Engineering, Beijing Institute of Technology, Beijing 100811, China
* Correspondence: lingyunyan@sit.edu.cn (L.Y.); bhxblz@bit.edu.cn (L.B.)

Table S1. Detailed Definitions of Spring Elements in Ligaments and the load transfers.

Ligament	Type of Spring Element	Stiffness (N/mm)	Function	Load Transfer (% of total joint load)
Posterior Cruciate Ligament (PCL)	Nonlinear spring element	450	Stabilizes knee joint, preventing hyperextension	10
Anterior Cruciate Ligament (ACL)	Nonlinear spring element	450	Prevents hyperflexion and controls anterior translation of tibia	8
Lateral Collateral Ligament (LCL)	Nonlinear spring element	300	Provides lateral stability, resists varus stresses	5
Medial Collateral Ligament (MCL)	Nonlinear spring element	300	Provides medial stability, resists valgus stresses	6
Meniscus Ligaments (horn attachments)	Nonlinear spring element	225	Facilitates load transmission through menisci	0

Table S2. Boundary Conditions for Varus-Valgus and Internal-External Rotation.

Condition Type	Flexion Angles (degrees)	Varus-Valgus Rotation (degrees)	Internal-External Rotation (degrees)
Initial Stance Phase	0 to 15	-5 to 5	-10 to 10
Mid-Stance Phase	15 to 45	-5 to 10	-10 to 15
Terminal Stance Phase	45 to 75	-10 to 5	-15 to 10
Swing Phase	75 to 0	-5 to 0	-10 to 0

Table S3. Results of sensitivity test.

Maximum contact pressure on femoral cartilage (Percentage change)				
Percentage change	Hamstrings force	Gastrocnemius force	Quadriceps force	Joint contact load
-20%	-3.55%	-3.14%	-4.01%	-14.75%
-10%	-2.28%	-1.76%	-3.14%	-9.40%
Baseline	0%	0%	0%	0%
10%	2.51%	-2.10%	2.74%	11.42%
20%	3.94%	-2.96%	3.92%	18.95%

Table S4. Differences of the maximum strain between lateral and medial cartilage across the whole phase.

	Strain of tibia cartilage	
Stance phase (%)	Medial	Lateral
0	0.0237	0.0198
5	0.0238	0.0201
25	0.0830	0.0687
50	0.0323	0.0343
75	0.0421	0.0407
100	0.0221	0.0180

Table S5. Root Mean Squared Error (RMSE) between our simulation results and experimental results.

RMSE between the simulation and experimental results [22,24]				
Parameter	Medial tibia contact area	Lateral tibia contact area	Medial tibia strain (%)	Lateral tibia strain (%)
RMSE	2.55	3.18	0.18	0.22
RMSE	2.71	3.04	0.21	0.24

Table S6. The maximum stress on meniscus with different Young's Modulus.

The varied E_{pt} and E_p(%)	Maximum stress (MPa)
-50	0.457
-40	0.508
-30	0.584
-20	0.759
-10	0.951
0	1.038
10	1.598
20	2.755
30	4.954
40	6.145
50	9.157

Table S7. Contact area on femoral and tibia cartilage (without patella joint).

	Tibia contact area (mm^2)		Femur contact area (mm^2)
Stance phase (%)	Medial	Lateral	Inferior
0	145	50	92
5	346	213	129
25	264	295	273
50	145	67	219
75	333	237	271
100	209	258	602

Table S8. Contact pressure on femoral and tibia cartilage (without patella joint).

	Tibia contact pressure (MPa)		Femur contact pressure (MPa)
Stance phase (%)	Medial	Lateral	Inferior
0	5.12	3.11	2.83
5	8.94	18.97	12.70
25	17.05	3.90	9.12
50	9.01	3.82	6.06
75	1.95	8.15	3.56
100	14.68	1.58	3.02

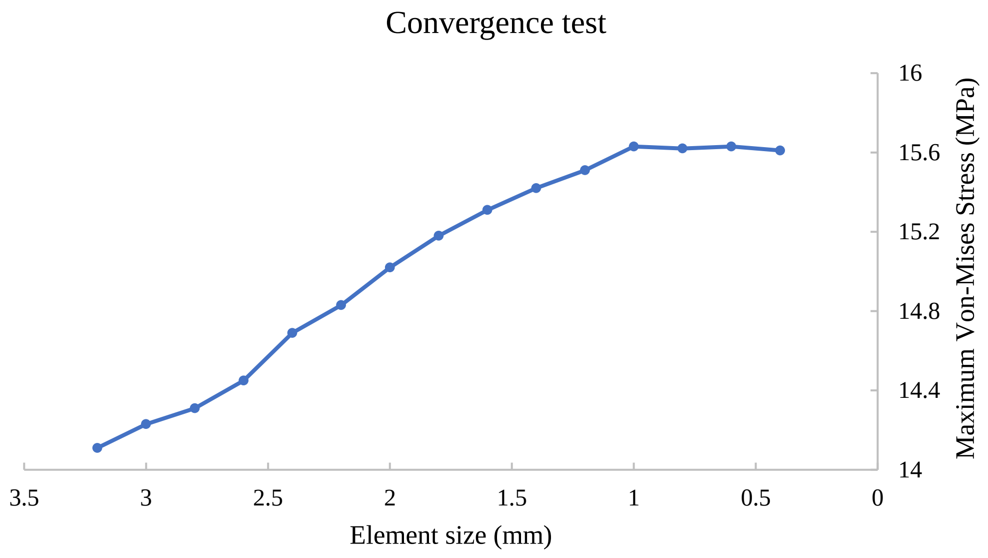

Figure S1. Size for all the tissues were modulated together with the cartilages.
